# Distanced Large Group Simulations as a Learning Method for Interprofessional Collaboration

**DOI:** 10.3390/nursrep14040195

**Published:** 2024-09-26

**Authors:** Marja Silén-Lipponen, Eija Piippo-Savolainen, Mina Azimirad, Terhi Saaranen

**Affiliations:** 1 Unit of Health Care, Savonia University of Applied Sciences, 70201 Kuopio, Finland; 2 Pediatrics Research Unit, Faculty of Medicine, University of Eastern Finland, 70210 Kuopio, Finland; eija.piippo-savolainen@pshyvinvointialue.fi; 3 Department of Pediatrics, Kuopio University Hospital, 70210 Kuopio, Finland; 4 Department of Nursing Science, Faculty of Health Science, University of Eastern Finland (UEF), 70211 Kuopio, Finland; mina.azimirad@uef.fi (M.A.); terhi.saaranen@uef.fi (T.S.)

**Keywords:** large group simulation, distance learning, interprofessional collaboration, healthcare education

## Abstract

Digitalization in healthcare education has shifted simulation learning methods to distanced implementations. Successful transition to distance education requires effective communication and the teacher’s good ability to use digital learning methods, as well as students’ active interaction and motivation throughout the entire educational process. This study explores participants’ experiences of online large group simulations as an approach for learning about interprofessional collaboration. A mixed method design was used in this study. Data from health and social care students and qualified professionals were collected using a questionnaire which contained statements presented on a five-point Likert scale and open-ended questions. The questionnaire was filled online by 100 students and professionals. The quantitative data were analyzed using descriptive statistical methods, and the open-ended questions were analyzed with inductive content analysis. As a result, the participants were satisfied with the large group simulation intended for learning interprofessional collaboration (mean = 4.42, SD = 0.759). The majority viewed it as a good learning method (95%) that is suitable for interprofessional collaboration learning (90%) and for promoting working together (82%). Digitally activated communication tools promoted interactive discussion and activated joint learning. However, only one activation tool was preferred instead of using two methods simultaneously. In conclusion, distanced large group simulations were evaluated to be a good method for learning interprofessional collaboration. This study was not registered.

## 1. Introduction

Reduced resources for organizing small group simulations cause challenges in health- and social care education [1]. In addition, the digitalization of healthcare and the global COVID-19 pandemic in 2019 forced educational institutions to switch, completely or partially, to distance learning, which increased the use of digital learning environments [2,3,4,5]. Some students have easily embraced the transition to distance learning and appreciated its flexibility in terms of geographic location and time. On the other hand, others have experienced discomfort due to limited digital literacy or a lack of physical human engagement and companionship [2,4,6].

In recent years, distance learning has been used to some extent because it enables learning in different situations and locations [7,8,9]. A large group simulation means that there are up to hundreds of participants at the same time, in contrast to a small group simulation where there are usually only 6–12 learners. Furthermore, in a large group simulation carried out remotely, there can be participants from several educational institutions and working life organizations. They take part in the joint event from locations of their own choice via their own remote device. Large group simulations have been performed using the hybrid method, with one group participating remotely and the other on site, which enables a twofold number of attendants in the learning session [8,10]. Additionally, large group simulations have consumed less time and simulation space than small groups simulations [11,12,13,14,15].

Use of the online delivery of simulation-based learning has necessitated the use of different activation methods to facilitate student engagement and active involvement [16]. In previous studies, the number of participants in large group simulations has varied from 15 to 427 [10,11,14,15,17,18]. The simulations have been implemented, e.g., as a play with pre-scripted situations and presented by either professional actors or qualified professionals in the field [15]. However, it can be challenging to maintain observers’ interest and engagement in simulation if they do not play an active role in the event [12,19]. Therefore, commitment to the simulation needs to be improved by several approaches, such as guided observation forms and online interaction with a chance to give feedback, ask questions, and suggest solutions to the situation [10,12,14,15,17]. Furthermore, reading the materials in advance and doing observer tasks during the simulation have been proven to be motivating and useful tools for students to reflect on their experiences from the perspective of learning outcomes [20,21].

The distance teaching implementations [22,23] have changed general teaching modalities, and therefore it has also been vital to make changes to the ways of activating students. So, more systematic approaches are needed to make the transition to distance education successful. Digitalization has helped to provide distance simulations [24], which require different preparations than face-to-face education, from both training providers and the participants [19,25,26,27]. The teacher’s capability to use digital learning methods ensures the logical progress of the simulation and helps students to maintain activating strategies [15,28,29]. For successful learning, students need motivation for distance learning, ability to monitor their own learning process, and suitable devices to participate online. In previous studies, students have emphasized the significance of fluent digital techniques, such as good visual and audio connections [19,24,30]. However, when the teaching mode changes from face-to-face learning to distance presentation, the students’ commitment in learning becomes unsure [4,14]. Further research is needed on students’ attitudes towards the use of online learning in healthcare [31], and especially in online simulation training [32]. At the moment, however, there are few studies on which special features of teaching and learning issues should be considered in remote simulations [18,19].

Interprofessional (IP) education has emerged as an important feature in education of health professionals, but it is often more time-consuming than traditional discipline-based education. However, IP healthcare education can be facilitated through various learning methods, including distance and large group simulations [5,19]. Large group simulations have demonstrated effectiveness in teaching IP skills [15,30] and have proven to be an effective approach to solidify the foundations for IP collaboration [10,14]. Despite two decades of simulation education, research into distance simulations, and especially IP skills learning, is still in its early stages [32]. Generating knowledge about learning experiences is important for advancing distance large group simulation as an innovative learning method for IP learning.

This study explored participants’ opinions and experiences of learning interprofessional collaboration in a distance simulation in a large group and posed the following two study questions.

1. What are the participants’ views on large group distance simulations in learning interprofessional collaboration?
2. How did the participants experience the large group distance simulation as a method for fostering interprofessional collaboration learning?

## 2. Materials and Methods

### 2.1. Study Context

The scenario manuscript for the large group simulation consisted of several video simulations and one live simulation. It was constructed by an IP group of qualified professionals, which included, among others, a pediatrician, social work teachers, nursing teachers, a physiotherapist teacher, a psychologist, and a high school teacher. In addition, the working group included two simulation learning and teaching researchers with extensive experience in simulation pedagogy. The patient scenario concerned a teen with excess use of painkillers and a threat of social exclusion due to functional back pain with a diverse variety of underlying causes.

The simulation was started with a brief introduction on prolonged pain in children and adolescents. Thereafter, the simulation proceeded by showing four video simulations. Each of them presented the patient’s situation from the perspective of different professionals, played by real-life practitioners. After a total of 25 min of the video simulations, a live simulation of IP care consultation on the patient’s problems took place in a lecture hall, followed by debriefing lasting roughly 40 min. All of the qualified professionals participating in the videos took part in the negotiation and in the final debriefing.

All the participants watched the video and live simulations livestreamed on their own smart device and participated in the events online. Online polling systems, Presemo™ and Mentimeter™, offered them a chance to vote or to give their written opinion after every video clip and during the negotiation and debriefing. Any anonymous comments made by the participants were displayed on a large screen in the lecture hall and were visible to everyone at the online event. The event ended with the summary of treatment options of functional prolonged pain in children and adolescents, presented by a hospital psychologist and a pediatrician.

### 2.2. Participants

An online link to the study questionnaire was sent via e-mail to all 275 persons who participated in the large group simulation. A total of 100 (36%) them, 78 university students from various courses and 22 working life healthcare professionals from different fields, gave their permission and answered the survey. The large group simulation was an optional course for all of the students and a voluntary in-service training for the professionals. The participants were mainly from pharmacy, dental, medicine, health sciences, psychology, and social sciences professionals and students from faculties in the University of Eastern Finland (*n* = 52) and social- and healthcare students from the Savonia University of Applied Sciences (*n* = 18) (Table 1).

### 2.3. Data Collection

The data collection for this study in October 2021 was based on a questionnaire (Large Group Interprofessional Simulation) that has previously been used in our large group simulation studies since 2017. The questionnaire consists of six background variables, 12 statements on a Likert scale of 1–5, and a few open-ended questions that have been developed and slightly modified according to the content of each large-scale simulation. The reliability of the measure has been verified by aligning the used Likert-scale statements to Cronbach’s alfa within the data of each annual survey [33].

### 2.4. Data Analysis

The quantitative data from the 12-statement questionnaires were analyzed statistically using IBM SPSS Statistics 27. Four of the questionnaire statements described the learning method, and their answers are reported in this article. At first, the initially given five-point-Likert scale answer options were reclassified into three categories (Table 2). Secondly, frequencies and descriptives were analyzed from the data (Table 1 and Table 2). In addition, the association between background variables and the answers were assessed using a cross-tabular analysis. The limit for statistically significant difference was *p* < 0.05.

**Figure 1:**
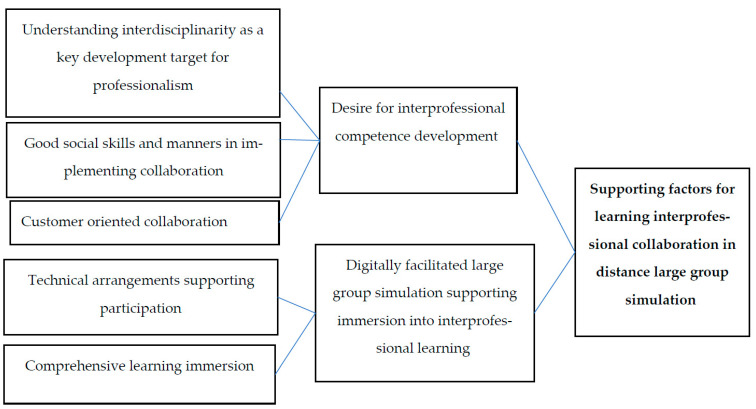
Factors supporting interprofessional collaboration in distanced large group simulations.

The qualitative data were analyzed using an inductive content analysis method. The intention of the content analysis is to describe the participants’ experiences by encoding and grouping systematically meaningful factors into categories and subcategories [33]. The analysis was carried out by reading the data through several times to find the insights meaningful to the research question. Thus, the original expressions reached were grouped into concepts related to the same category. By comparing the concepts with each other and further analyzing and intensifying them, the subcategories of the analysis were produced. The subcategories were further analyzed, and ultimately two main categories describing the participants’ experiences of the supporting factors for learning interprofessional collaboration in distanced large group simulations were named (Figure 1).

### 2.5. Ethics

This study was designed and performed according to the Finnish law and ethical declarations and approved by the Research Ethics Committee of the University of Eastern Finland (statement June 2016 and updated October 2023). Before the simulation, the participants were provided with written information about the simulation and the study. The study aim, confidentiality, and voluntary participation were explained in the questionnaire. The confidentiality was ensured by excluding all personal data of the participants, and the study data were stored in a protected cloud storage service according to the university’s guidelines. This study followed the General Data Protection Regulation (Regulation EU, 2016).

## 3. Results

### 3.1. Background Information

Most of the participants were female (91%) and younger than 40 years old (63%) (Table 1). Many of the participants (72%) had work experience in social care and healthcare professions, with most of them reporting having the nursing occupation (35%), followed by healthcare sciences (24%).

### 3.2. The Participants’ Views of Distance Large Group Simulation in Learning Interprofessional Collaboration

Generally, the participants were satisfied with the large group simulation for learning IP collaboration (mean = 4.42, SD = 0.759). The majority viewed it as a good learning method (95%), suitable for IP learning (90%), promoting working together after working life training (82%), and encouraging participants to be active (79.8%). Participants’ views on the large group simulation did not differ significantly according to their background information (Table 2).

### 3.3. Distance Large Group Simulation as a Method for Fostering Interprofessional Collaboration Learning

The results of the qualitative analysis consisted of two main-categories: desire for IP competence development and digitally facilitated simulation supporting immersion into IP learning (Figure 1), which promoted collaboration in the distance large group simulation.

### 3.4. Desire for Interprofessional Competence Development

The large group simulation strengthened participants’ understanding of interdisciplinarity as a key development target for professionalism. In addition, it encouraged both the students and the professionals to maintain a reciprocal and open learning atmosphere. The simulation clarified the knowledgebase of the work of various professional groups and confirmed the appreciation of both their own and others’ work. The relevance of interdisciplinarity became visible by understanding the fact that no one needs to solve the patients’ challenging situations alone. Instead, anyone can learn interdisciplinary collaboration by having trust in all of the professionals’ capacities to produce expertise in the care.

*During our studies, we have worked only a little in cooperation with students from other professions, so the large-group simulation gave an idea of the importance of cooperation.*

*It is important to see the experts in different fields pulling in the same direction and respect each other’s professional skills. Hopefully, I know how to do so at work!*

*No one must know about everything but remember to make use of the expertise of others.*

From the participants’ point of view, interdisciplinarity was supported in the simulation by good social skills and manners. Each professional group approached the patient’s situation from various perspectives, even though their aim was common. The honest collaboration was based on fluent information flow and the possibility to share opinions of the patients’ care within the interprofessional group.

*Multi-professional working is hampered if there are professionals with defective manners, or who are not ready to see other kinds of perspectives on the patient’s situation.*

*It is a wealth that professionals look at the patient’s situation from a different angle, even though everyone has the same goal in the final games.*

The simulation strengthened patient-centered collaboration. In addition to talking, active listening of both patients and colleagues was needed to overcome the unilateral aspects and to concentrate on the most significant problems. It was not completely clear to whom and what kind of patient data are allowed to be shared in an interdisciplinary social- and healthcare team. The simulation strengthened the understanding of the fact that a young patient’s consent is needed for sharing their information with other professionals and even with their family.

*Focusing on the patient is important. I take many insights from the simulation instantly into my own work!*

*After participating in so many multidisciplinary meetings, I will continue to pay even more attention to the patient’s involvement.*

The digitally facilitated large group simulation supported immersion into interprofessional learning.

Good technical arrangements of the large group simulation enabled a substantial number of people to participate remotely in the same learning situation with their own electronic device. The PRESEMO™ and Mentimeter™ Digital Activation Tools promoted interactive discussion and activated joint learning. Furthermore, digital conversation brought a deep sensation for the participants to be an integral part of the shared experience. In addition to activating tools, the orientating videos maintained an interesting learning atmosphere and responded to the questions and thoughts participants’ had during the event.

*Activating auxiliary systems and technology played nicely!*

*In active intervals the inclusive queries kept up the interests.*

Only one activation tool would have been preferred, instead of using two methods that drew attention from the content of the simulation. Using the two activation tools at the same time would have required a clearer login for the platforms. In this simulation, the login for the other platform was needed repeatedly, and the code was changing several times. This made it difficult to use that platform.

*A couple of times I left without writing comments when I didn’t have time to get the code.*

*It would be clearer to have just one place for surveys and comments. The use of the Mentimeter was inconvenient because it had to be logged every time again with a different code.*

The distance learning large group simulation formed a comprehensive learning experience. It enabled participants to have a focused and intimate involvement. It was easier to comment remotely than it was in the big auditorium where everyone was listening or speaking.

*Presemo contained a variety of ideas and anonymity was working in this situation.*

*At home I was able to focus on peace at the events and did not need to be thrilled!*

A pleasant and acceptable atmosphere reached the audience at both the emotional and educational levels, and thus promoted learning. On the other hand, some participants would have hoped for a longer-lasting interdisciplinary meeting, because it was an educational and authentic possibility to follow.

*Videos worked well on this topic. Good pedagogy behind!*

*It was nice when you got to participate in surveys and voting electronically at home.*

During the interdisciplinary meeting, participants did not always remember each member’s original profession, which weakened learning immersion and the understanding of decision making. Some would have wanted to participate in the debriefing in a face-to-face meeting because they thought that it would have offered them a better time to monitor the simulation. It was suggested that participants had access to at least some of the simulation videos beforehand to include more time for the discussion.

*Communality suffers when everyone is distant. I assume we would have learned more if we students also could have spoken normally.*

*Occasionally the pace was fast, and you felt that you did not have time to follow the videos, answer the one-line questions and, for example, write notes.*

## 4. Discussion

This study provides new descriptive insights into interprofessional (IP) learning by utilizing a large group simulation as a distance learning approach in healthcare education. Large group simulations offer an alternative to small group simulations, allowing for many participants to practice challenging IP scenarios together. These scenarios are designed to involve interactions without clear-cut solutions. In a large group simulation, participants learn by observing actors and reflecting on the objectives under the guidance of facilitators.

The primary finding indicates that the distance IP large group simulation effectively facilitates the acquisition of IP collaboration competencies and can either add or replace the traditional classroom simulation. Both quantitative and qualitative results align with prior research, confirming that large group simulations are a suitable method for IP learning [10,14,15,30].

In the present study, the distanced large group simulation was found to be an effective and stimulating way to learn IP skills, supported by good manners and a patient-centered caring approach. This observation is in line with previous studies that have shown the possibility to also learn IP collaboration skills online [9], and even in large groups with up to 250 attendants [34]. Furthermore, large group simulations provide cost benefits when teaching is conducted remotely and recorded, allowing for students to return to the content multiple times. This method works synergistically with other educational resources that utilize technical resources [35].

Distance connections offer a modern way to expand simulation training beyond the classrooms and to reach large numbers of attendants simultaneously. They are dependent on good technical devices that allow for students to interact with instructors in real time, leading to deeper learning and saving time, effort, and money [9,18,36]. In line, the skillful technical implementations were considered vital for a successful learning experiences in the present study. Most importantly, distanced simulations seem to produce authentic learning experiences [30] that are comparable to those in real life simulations [37,38].

Simulation learning is based on the positive connection between emotions and learning [39]. However, it is also known that classroom and distance simulations may cause stress and nervousness [40,41,42,43], resulting in learning impairment [39]. Based on our results, it seems that online participation in simulations alleviated the attendants’ anxiety and offered a non-stressing way to actively take part in the collaborative connection with others. It seemed to promote learning important skills for interprofessional work, such as trusting others know-how, sharing information, and being reflective to others’ opinions.

In our research, the students’ own desire to learn collaboration was an important promoter of learning. Both the learners’ engagement and satisfaction with the learning process have been of utmost importance in previous studies as well [44,45]. The individual learning style and the learning environment affect the learning experience, and therefore multiple ways of maintaining the attendants’ interest are needed. In the present study, the participants highly valued the possibility of being online and using activating tools that made the event interactive and inspiring. All of the participants were university students or qualified professionals who had graduated from university. Therefore, all of the participants had knowledge about the topics of the simulation produced by their education. In addition, they had been sent orientating learning material in advance to familiarize themselves with the topic before the simulation. In previous studies, small group conversations [9], well-targeted pre-reading materials [46], and clear observer tasks during online simulation have deepened the perceptions of participants and thus enhanced IP collaboration [46,47]. Furthermore, post-simulation reflective essays, that were also included in the present simulation for students of some disciplines, have been shown to improve the learning results as well [48].

The importance of feedback, debriefing, and self-debriefing has been emphasized as promoting effective learning in simulations [49]. In remote simulations, methods such as personally guided debriefing sessions, self-examining questions, and synchronous virtual debriefing have been useful for learning [49,50]. In this study, the debriefing was carried out online, and the learners had the opportunity to participate to the conversation on their own mobile devices remotely. This increased reflection and deepened IP knowledge. In addition, when the participants had the opportunity to interact with students and professionals from other professional fields, they learned more comprehensively about their own and others’ roles and responsibilities [5,31]. It appeared that students have extensive recent experience with online learning and the increase in technological solutions in healthcare education in general. For example, experiences during the COVID-19 pandemic increased students’ ability to use technology in distance learning [35]. Sharing written comments online has been found to be a visual and illustrative way to express personal opinions and create a sense of active participation [25]. Based on the qualitative data, many of the learners appreciated the methods used in this simulation. However, some of them expressed their preference for face-to-face teaching. Therefore, technology could be used in a more versatile way than at present to activate participants, for example, by more effective use of chat conversations.

In the present study, confidential and warm interaction between the professionals, as well as between the customer and the professionals, was found to be an important promoter for successful IP teamwork. The simulation gave the participants a good insight into functioning teamwork and encouraged them to contact professionals from other fields of expertise, too. In line with this finding, a positive learning atmosphere has been found to promote successful IP problem solving and to be an important basis for safe simulation learning when practicing the empathetic and customer-centered encounter needed in the health sector [42].

### Strengths and Limitations

The data collection was performed reliably online by our previously constructed questionnaire with minor modifications for the present study. Different question types, both structured and open-ended questions, supplemented with quotes, complemented the knowledge [26]. As a limitation, no questions on factors restricting learning in the large group simulation were presented. The uneven number of participants did not allow for the comparison of learning experiences and opinions between different professional groups. In addition, there was no arranged possibility for small group conversations, which might have deepened the students’ insights to the case. However, our results suggest that distanced large group simulations could be a cost-effective method in IP education along with other learning methods.

## 5. Conclusions

Based on the present study, distance learning in a large group simulation strengthened a comprehensive understanding of both interdisciplinarity and patient-centered collaboration. The modern digital methods, like video simulations, online polling tools, and the possibility to take part in the conversation remotely, were successful means for maintaining the attendants’ interest and commitment to the simulation. Although technical execution was successful for most of the participants, the complexity of technical methods or timely limitation of the schedule might have restricted optimal learning experiences. The scenarios developed in this study succeeded in producing a unique IP learning experience, in which nearly 250 persons participated simultaneously. Such distanced large group simulations can also most likely be used to learn other topics in a cost-effective way.

## Figures and Tables

**Table 1:** Background information (*n* = 100).

	*n* (%)	Mean (SD)	Min–Max
Gender	100 (100%)		
Female	91 (91%)		
Male	7 (7%)		
Other	0 (0%)		
I don’t want to say	2 (2%)		
Age (year)	99 * (100%)	36.11 (10.62)	20–63
Below 39	63 (63%)		
40–49	25 (25%)		
50–59	9 (9%)		
60 or more	3 (3%)		
I participate in the study as a	100 (100%)		
Student	78 (78%)		
Representative of working life	22 (22%)		
Education Organization	82 ** (100%)		
University	52 (63.4%)		
University of Applied Sciences Other	18 (21.9%) 12 (14.7%)		

* missing data: *n* = 1. ** missing data: *n* = 18.

**Table 2:** Summary of the aggregate responses to the survey (*n* = 100).

Statements	*n* (%)			Mean (±SD)
	Disagree *	Neither Agree nor Disagree	Agree **	
Large group simulation was a good learning method for learning interdisciplinary collaboration.	2 (2%)	3 (3%)	95 (95%)	4.63 (±0.646)
Interdisciplinary large group simulation was a learning method that made participants active.	5 (5%)	15 (15%)	79 (80% ***)	4.15 (±0.930)
Large group simulation as a distance learning method was suitable for interdisciplinary learning.	2 (2%)	8 (8%)	90 (90%)	4.45 (±0.730)
Interdisciplinary large group simulation encourages working together even after training in working life.	3 (3%)	5 (5%)	92 (92%)	4.47 (±0.731)

Disagree * = completely disagree and partially disagree, agree ** = partially agree and completely agree. *** the answer to this question was missing from one participant (*n* = 99).

## Data Availability

The data presented in this study are available on request from the corresponding author. The data are not publicly available due to keep the confidentiality.
